# Lutein-in-Extra-Virgin-Olive-Oil Supplementation as a Multifaceted Nutritional Intervention for Retinopathy of Prematurity: Evidence from the Mouse Oxygen-Induced Retinopathy Model

**DOI:** 10.3390/nu18213616

**Published:** 2026-10-30

**Authors:** Xiaoyuan Ye, Lu Xiang, Ka Yee Chen, Leo O Lai, Jetty Chung-Yung Lee, Antonia M. Joussen, Wai-Ching Lam, Amy C. Y. Lo

**Affiliations:** 1 Department of Ophthalmology, LKS Faculty of Medicine, The University of Hong Kong, Hong Kong 999077, China; yexy9@connect.hku.hk (X.Y.);; 2 School of Biological Sciences, The University of Hong Kong, Hong Kong 999077, China; 3 Department of Ophthalmology, Charité—Universitätsmedizin Berlin, 12203 Berlin, Germany; 4 Department of Ophthalmology, Vancouver General Hospital, University of British Columbia, Vancouver, BC V5Z 3N9, Canada

**Keywords:** ischemic retinopathy, carotenoid, inflammation, blood-retinal barrier (BRB), neovascularization, metabolites

## Abstract

Background: Retinopathy of prematurity (ROP) is a predominant contributor to preventable childhood blindness driven by oxidative stress and inflammation, but with limited treatment options. Safe, non-invasive adjunctive strategies become attractive. Lutein displays antioxidant and anti-inflammatory properties, but its clinical efficacy remains controversial. We proposed that formulating lutein in extra-virgin olive oil (EVOO) might enhance its absorption and efficacy in ROP. Here, we evaluated the effects of lutein-in-EVOO (LEVOO) in oxygen-induced retinopathy (OIR), a mouse model of ROP. Methods: C57BL/6J mouse neonates received daily gavage of LEVOO (0.4 μg/g, 1.8 μg/g and 3.6 μg/g of lutein in EVOO) after OIR challenge from postnatal day (P) 12 to P16. Assessments were conducted at P17, including retinal vasculature, function, cytoarchitecture, inflammation, and oxidative damage; serum biochemical and antioxidant profiles; and proteomic analysis and targeted retinal metabolomic analysis. Results: Oral supplementation of LEVOO was well-tolerated and safe in mouse neonates. It reduced retinal avascular and neovascular areas, increased electroretinographic b-wave amplitudes, and restored amacrine cells and rod bipolar cells. LEVOO administration reduced oxidative damage and activated microglia. LEVOO administration inhibited the hypoxia-inducible factor-1α (HIF-1α)/macrophage migration inhibitory factor (MIF) signaling and low-density lipoprotein receptor-related protein-1 (LRP1)-dependent cyclophilin A (CypA)/matrix metalloproteinase-9 (MMP9) signaling. It also helped to partially restore retinal docosahexaenoic acid and dopamine in OIR mice. Conclusions: LEVOO demonstrated multifaceted efficacy in OIR. It reconstructed retinal vasculature, restored retinal function, and rescued neuronal cell loss with strong antioxidative and anti-inflammatory effects. Collectively, these findings establish LEVOO as a promising non-invasive complementary strategy for ROP.

## 1. Introduction

Retinopathy of prematurity (ROP) is a retinal vaso-proliferative disease in preterm and low-birth-weight infants, contributing to childhood blindness worldwide [1]. ROP prevalence varies by level of neonatal care, from 5–8% in developed countries to up to 30% in middle-income developing countries [2,3].

The pathophysiology of ROP is linked to the immaturity of the retinal vasculature at birth in preterm infants and to the subsequent need for oxygen therapy, with a biphasic course [4]. In Phase I, the hyperoxic environment suppresses the production of pro-angiogenic factors like vascular endothelial growth factor (VEGF) and elevates reactive oxygen species (ROS) levels, thereby preventing normal vessel growth. In Phase II, the cessation of supplemental oxygen induces relative hypoxia in these avascular zones, triggering compensatory but pathological neovascularization. These are fragile and disorganized vessels that are easy to induce vitreous hemorrhage, retinal detachment, and ultimately, blindness [5,6].

Available treatments for ROP consist of laser photocoagulation, cryotherapy, and intravitreal anti-VEGF injections, which are inherently invasive and carry significant limitations and risks [7,8,9]. Consequently, there is a need to develop adjunctive or preventive management for ROP that is safe, non-invasive, and effective.

Nutraceuticals, as bioactive dietary supplements, have emerged as attractive candidates for ROP intervention due to their generally favorable safety profiles [10]. Among them, lutein, a xanthophyll carotenoid highly concentrated in the human macula, has attracted considerable interest. Lutein is synthesized naturally by plants and photosynthetic microorganisms. Humans are incapable of *de novo* lutein biosynthesis and must obtain lutein entirely through dietary or supplemental intake. Lutein has antioxidative and anti-inflammatory properties, with studies reporting its role in protecting photoreceptors, maintaining visual function, and mitigating oxidative stress [9,11]. Our previous preclinical work also demonstrated that intraperitoneal administration of lutein effectively promoted normal retinal vascular regeneration in a mouse model of ROP [12].

However, translating the promising preclinical findings into consistent clinical benefit has proven challenging. Recent randomized controlled trials studying lutein’s effects in ROP preterm infants reported that lutein did not significantly prevent the onset of proliferative ROP [13,14,15]. Yet macular carotenoid supplementation (including lutein) may not lower the total incidence of ROP, but it might help prevent progression to more severe stages [16]. A key factor underlying these translational discrepancies is the low and variable oral bioavailability of lipophilic lutein. Therefore, improving the delivery and bioavailability of lutein is a critical step toward re-evaluating its therapeutic efficacy for ROP.

Co-ingestion with dietary fat enhanced lutein’s intestinal absorption [17]. Olive oil (OO) naturally contains lutein, while its concentration varies. OO outperformed other vegetable and fish oils in enhancing lutein absorption, likely due to the enrichment in oleic acid [17,18,19]. Importantly, OO-based emulsions have demonstrated good tolerability in nutritional support for preterm infants, providing a crucial safety rationale for their use in this vulnerable population [20]. Extra-virgin olive oil (EVOO), obtained through mechanical extraction, retains a high concentration of natural antioxidants. EVOO itself possesses established independent positive wellness impacts, such as cardiovascular benefits, anti-inflammatory potential, and lipid metabolic modulation [21,22]. Previous studies have demonstrated that EVOO protected against endothelial dysfunction and inhibited VEGF-induced angiogenesis, suggesting that EVOO may also be beneficial in ROP [23,24]. These findings provide a rationale for selecting EVOO as the vehicle for lutein in the context of ROP.

Here, we hypothesized that formulating a lutein supplement suspended in EVOO (lutein-in-extra-virgin-olive-oil, LEVOO) may provide benefits for infants with ROP. We employed the oxygen-induced retinopathy (OIR) model to evaluate the safety and efficacy of oral LEVOO supplementation in neonatal mice, thereby preclinically assessing LEVOO’s potential as an adjunctive nutritional therapy.

## 2. Materials and Methods

### 2.1. Animals and the OIR Model

**Figure 1:**
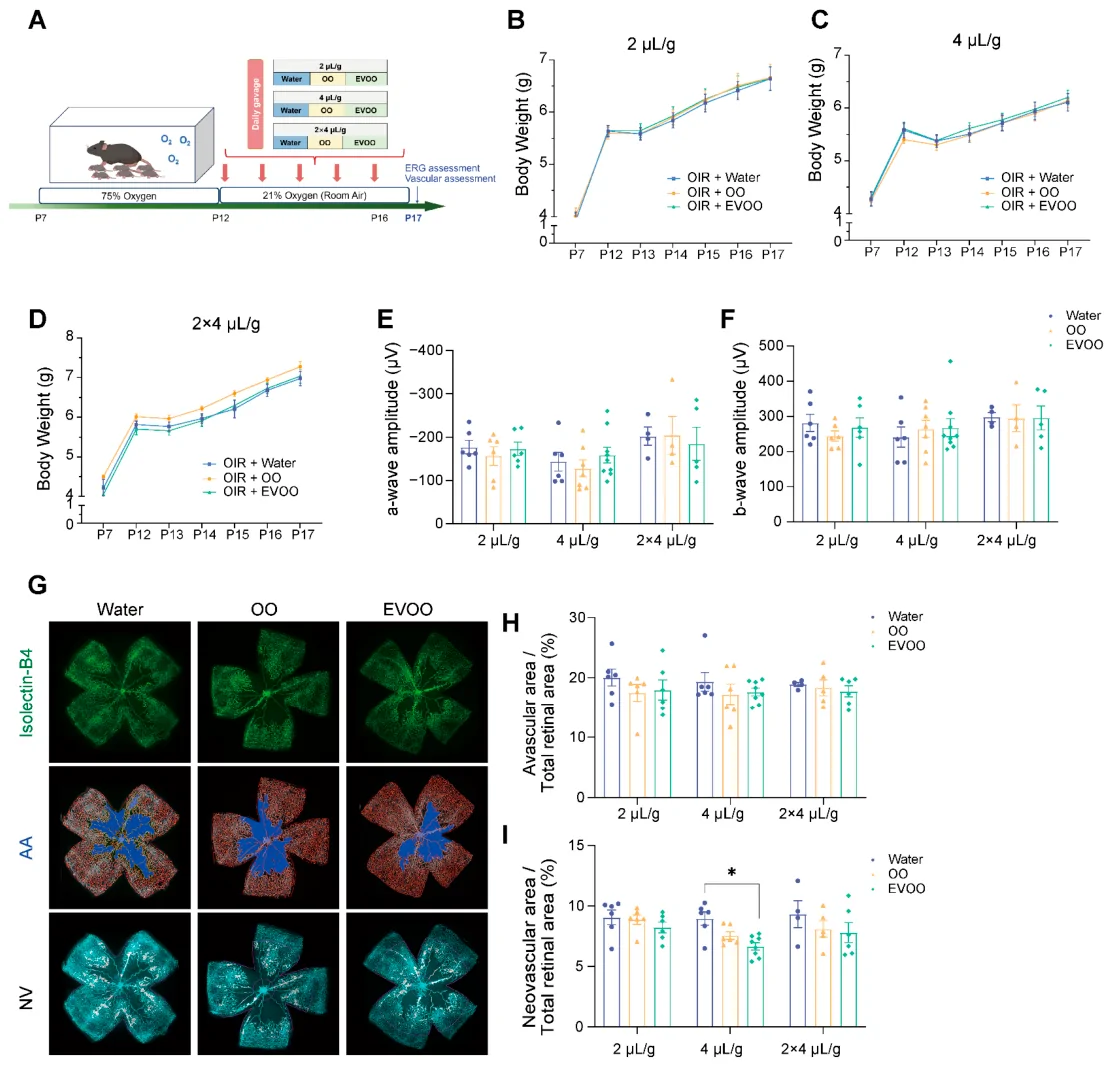
EVOO at a dosage of 4 μL/g alleviated neovascularization in OIR. (**A**) Schematic graph showing the experimental timeline. (**B**–**D**) Body weight of neonates. (**E**,**F**) Analysis of scotopic ERG [a-wave amplitudes (**E**) and b-wave amplitudes (**F**)] at P17. (**G**) Representative images of isolectin-stained retinas and the analyzed images [avascular area (AA, blue) and the neovascular area (NV, white)]. (**H**,**I**) The quantitative analysis of AA and NV (*n* = 3–9, one-way ANOVA followed by Tukey’s post hoc test).

**Figure 2:**
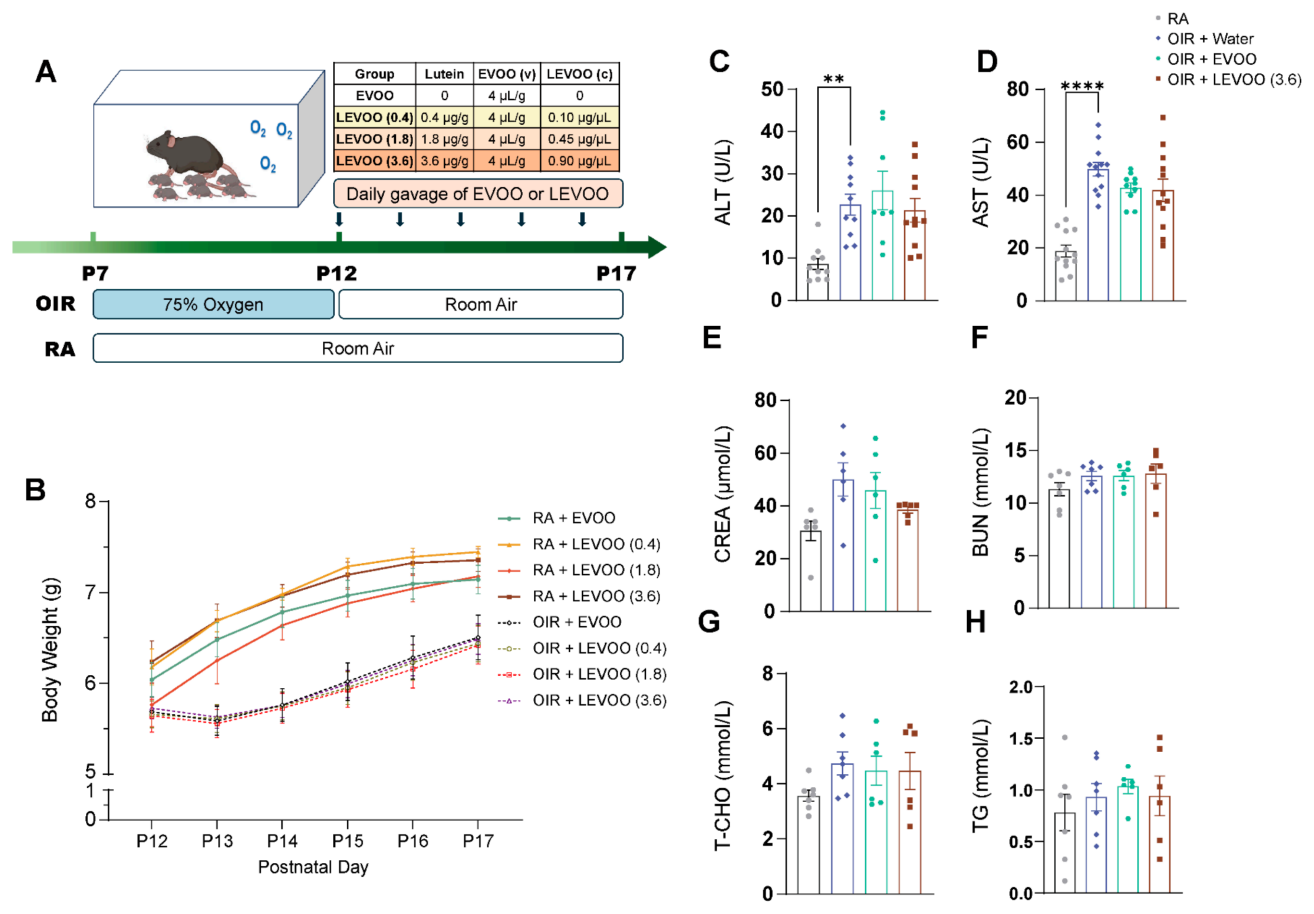
Oral supplementation of LEVOO was safe for neonatal mice upon OIR challenge. (**A**) Schematic diagram showing the experimental timeline. The volume (v) administered was 4 μL/g, and different concentrations (c) of LEVOO were prepared. (**B**) Body weight of neonates in various experimental groups. (**C**–**H**) Serum biochemical levels of neonates raised in RA or upon OIR challenge and fed with water, EVOO or LEVOO with a lutein dose of 3.6 μg/g (*n* = 6–12; one-way ANOVA followed by Tukey’s test).

Wild-type C57BL/6J mice were housed under controlled temperature with *ad libitum* food and water. The OIR model was induced as we published earlier [12]. Briefly, while still nursed by their mothers, mouse neonates were put in a chamber (PRO-OX110, BioSpherix Ltd., New York, NY, USA) containing 75% O_2_ from postnatal day 7 (P7) to P12 and were put back to room air afterward (Figure 1A and Figure 2A). In this study, the number of neonates per litter was limited to 6 to ensure that all neonates in the cage had adequate nutrition [25]. Only neonates weighing between 5.5 and 7.5 g on P17 were included [25,26].

### 2.2. EVOO and Lutein Administration

Supplementation was administered daily by the intragastric route from P12 to P16. Various volumes (2 μL/g, 4 μL/g, or 2 × 4 μL/g, each twice the preceding dose) of EVOO (Carbonell^®^, Carbonell Cia. Anónima, Rubí, Spain), ordinary OO, and water were administered to OIR mice (Figure 1A). Water and OO served as volume control and caloric control, respectively. For 2 × 4 μL/g, administration was divided into two daily feedings of 4 μL/g to avoid an excessively large gavage volume for neonatal mice. A total of 4 μL/g EVOO was eventually selected as the optimal administration volume.

Lutein (PHR1699, Sigma-Aldrich Corp., St. Louis, MO, USA) was suspended in EVOO to prepare an LEVOO suspension and adjusted to a volume dose of 4 μL/g. Lutein has been reported to be safe without serious side effects at 20 mg/day in humans [27,28]. A total of 2 mg/day is one order of magnitude lower. Meanwhile, 10 mg/day was used in the AREDS2 formula [29]. These human dosages (2 mg/day, 10 mg/day and 20 mg/day) were therefore selected and converted to the mouse equivalent dose (MED) according to the formula [30]:

MED (mg/kg/day) = human dose (mg/kg/day) × mouse Km ratio (Km ratio for mouse = 12.3)

Thus, doses of 0.4 μg/g/day [LEVOO (0.4)], 1.8 μg/g/day [LEVOO (1.8)] and 3.6 μg/g/day [LEVOO (3.6)] were used (Figure 2A).

### 2.3. Retinal Function Analysis by Electroretinogram (ERG)

After overnight dark adaptation, P17 mouse neonates were anesthetized with an intraperitoneal ketamine (0.6 mg/100 μL/10 g)/xylazine (0.15 mg/100 μL/10 g, Alfasan International, Woerden, The Netherlands) mixture. Tropicamide/phenylephrine hydrochloride eye drops (0.5%, Mydriacyl^®^, SANTEN OY, Tampere, Finland) were used for pupil dilation. Proparacaine hydrochloride (0.5%, ALCAINE^®^, SA ALCON-COUVREUR NV, Puurs, Belgium) was used for topical anesthesia. ERG was performed using the ColorDome Ganzfeld System (Diagnosys, Lowell, MA, USA) [31].

### 2.4. Immunostaining of Retinal Whole-Mounts

Eyes were enucleated and fixed with 4% paraformaldehyde (PFA) (4 °C, 100 min). The retina was isolated, cut and flattened into a “petal” shape to create retinal flat mounts. The retinal whole-mounts were first probed with primary and then secondary antibodies (Table S1). Subsequently, they were transferred onto glass slides, cover-slipped, and imaged using fluorescence microscopy (Eclipse 80i, Nikon, Tokyo, Japan). Images were merged using ImageJ (version: 1.53t).

The avascular area (AA) and neovascular area (NV) of the retinal flat mount were evaluated using the open-source MATLAB (version: R2022b) tool [32]. Percentages of AA and NV were calculated as follows.

avascular area/total retinal flat mount area × 100%

neovascular area/total retinal flat mount area × 100%

Immunostained retinal flat mounts were also used to analyze endothelial tip cells. Three to four images of retinal vessels in the transition zone between the avascular and vascular regions were captured (Eclipse 80i, Nikon, Tokyo, Japan). Tip cells were counted independently and blindly by two observers. For microglia analysis, Z-stack confocal images of central (avascular zone) and peripheral zones (with tufts or without tufts) within the superficial vascular plexus were collected using a confocal microscope (LSM 900, Zeiss, Germany). The number of microglia in 4–6 selected 200X fields was counted.

### 2.5. Paraffin Embedding of Eyeballs

Eyeballs were fixed with 4% PFA and embedded in paraffin. Sagittal sections (5 μm thick) with the optic nerve head were made and used for hematoxylin and eosin (H&E), immunofluorescence (IF) and immunohistochemical (IHC) staining.

### 2.6. IF Staining

IF staining was performed as described earlier [33] (antibodies listed in Table S1). Images were acquired using a fluorescence microscope (LSM 900, Zeiss, Jena, Germany). Mean fluorescence intensity values were measured using the built-in ZEN software (2012 blue edition). Calretinin-positive cells in the innermost inner nuclear layer (INL) were counted per visual field [33]. Tyrosine hydroxylase (TH)-positive cells were quantified in whole sections.

### 2.7. IHC Staining

IHC was performed using antibodies listed in Table S1. Staining was visualized with a DBA kit (ZLI-9018, Beijing ZSGB-Bio, Beijing, China). Images were obtained with a microscope (Eclipse 80i, Nikon, Tokyo, Japan), and the immunoreactivity was quantified using the ImageJ plugin, IHC Toolbox.

### 2.8. Serum Collection and Biochemical Measurement

Blood was drawn via cardiac puncture immediately after euthanasia and allowed to clot. After centrifugation, the supernatant (serum) was obtained for assays of total superoxide dismutase (SOD), reduced glutathione (GSH) and total antioxidant capacity (T-AOC), as well as liver and kidney function markers (Table S2). Retinal tissues were lysed and prepared according to the protocols of the assay kits.

### 2.9. Mouse Retinal Metabolite Assessment

Six retinas from three mice were pooled. Targeted quantification of polar metabolites and C4:0-C26:0 fatty acids via GC-MS/MS was conducted at the Center for PanorOmic Sciences, The University of Hong Kong. Agilent MassHunter software (v.13.0) was used for data analysis. Principal component analysis (PCA) was performed on the identified fatty acid composition. The PCA biplot and the heatmaps (based on Z-score) were generated using the online open-source tool Wei Sheng Xin [34].

### 2.10. Proteomics Analysis

Proteomic analysis was performed using pooled samples from 4 retinas of two mice and entrusted to BGI Genomics (HK). Data were analyzed and graphs were generated using the Dr. Tom online analysis platform (BGI Genomics Co., Ltd., Shenzhen, China).

### 2.11. Western Blotting (WB) Analysis

Antibodies for WB analysis were listed in Table S1. ImageJ was employed to quantify the band intensities.

### 2.12. Enzyme-Linked Immunosorbent Assay (ELISA)

ELISAs for mouse VEGF-A, mouse interleukin-1β (IL-1β), and mouse IL-10 were conducted based on the manufacturer’s instructions (Table S2).

### 2.13. Statistical Analysis

Data were presented as mean ± standard error of the mean (SEM). Normally distributed data were analyzed with an unpaired *t*-test or one-way ANOVA, followed by Tukey’s test or Dunnett’s post hoc test. Data were analyzed and visualized via GraphPad Prism 10. A *p*-value < 0.05 was considered statistically significant (*, *p* < 0.05; **, *p* < 0.01; ***, *p* < 0.001; ****, *p* < 0.0001). The absence of significance annotations on the plots indicated that no statistically significant difference existed between the groups compared.

## 3. Results

### 3.1. EVOO at 4 μL/g Alleviated Neovascularization in OIR

Various volumes (2 μL/g, 4 μL/g, or 2 × 4 μL/g) of EVOO, ordinary OO, and water were administered to OIR mice (Figure 1A). As body weight can affect pathological vascular outcomes in OIR [26], water and OO were used as volume control and caloric control, respectively. Body weight monitoring revealed no significant differences among the three administration groups across all gavage volumes, suggesting that OO or EVOO administration did not induce additional weight gain or weight loss (Figure 1B–D). Furthermore, ERG measurements showed no significant differences in a-wave or b-wave amplitudes among groups (Figure 1E,F), indicating that EVOO administration did not impair retinal function in OIR mice.

Regarding retinal vasculature, EVOO alone tended to reduce neovascular areas (NV) and avascular areas (AA) when compared with water at the dose of 2 μL/g or 2 × 4 μL/g, although these effects were not statistically significant (Figure 1G–I). EVOO-treated mice also showed a trend of reduced neovascular area compared with OO-treated mice across all feeding doses (Figure 1G,I). Notably, a significant reduction in neovascularization was shown in EVOO-fed mice at 4 μL/g when compared with the water-treated group (Figure 1G,I). Therefore, the dose at 4 μL/g was selected as the optimal volume for delivering lutein in the LEVOO suspension.

### 3.2. Oral Supplementation of LEVOO Was Safe for Neonatal Mice upon OIR Challenge

LEVOO supplementation did not change the body weight of mice reared in normal room air (RA) or with OIR challenge (Figure 2A,B). To further investigate the potential systemic effects of EVOO and LEVOO, we analyzed serum biochemical markers of hepatic and renal function, including aspartate aminotransferase (AST), alanine aminotransferase (ALT), triglycerides (TG), total cholesterol (T-CHO), blood urea nitrogen (BUN) and creatinine (CRE). While the OIR challenge itself elevated ALT and AST levels, these remained within the normal physiological range in adult C57BL/6J mice [35]. Notably, mice treated with EVOO or LEVOO (with a lutein dose of 3.6 μg/g) exhibited similar levels of ALT, AST, TG, T-CHO, CRE, and BUN, indicating that oral administration of EVOO or LEVOO did not induce hepatotoxicity or nephrotoxicity, thus confirming their safety profile (Figure 2C–H).

### 3.3. LEVOO Administration Improved Vascular Health in OIR Mice

**Figure 3:**
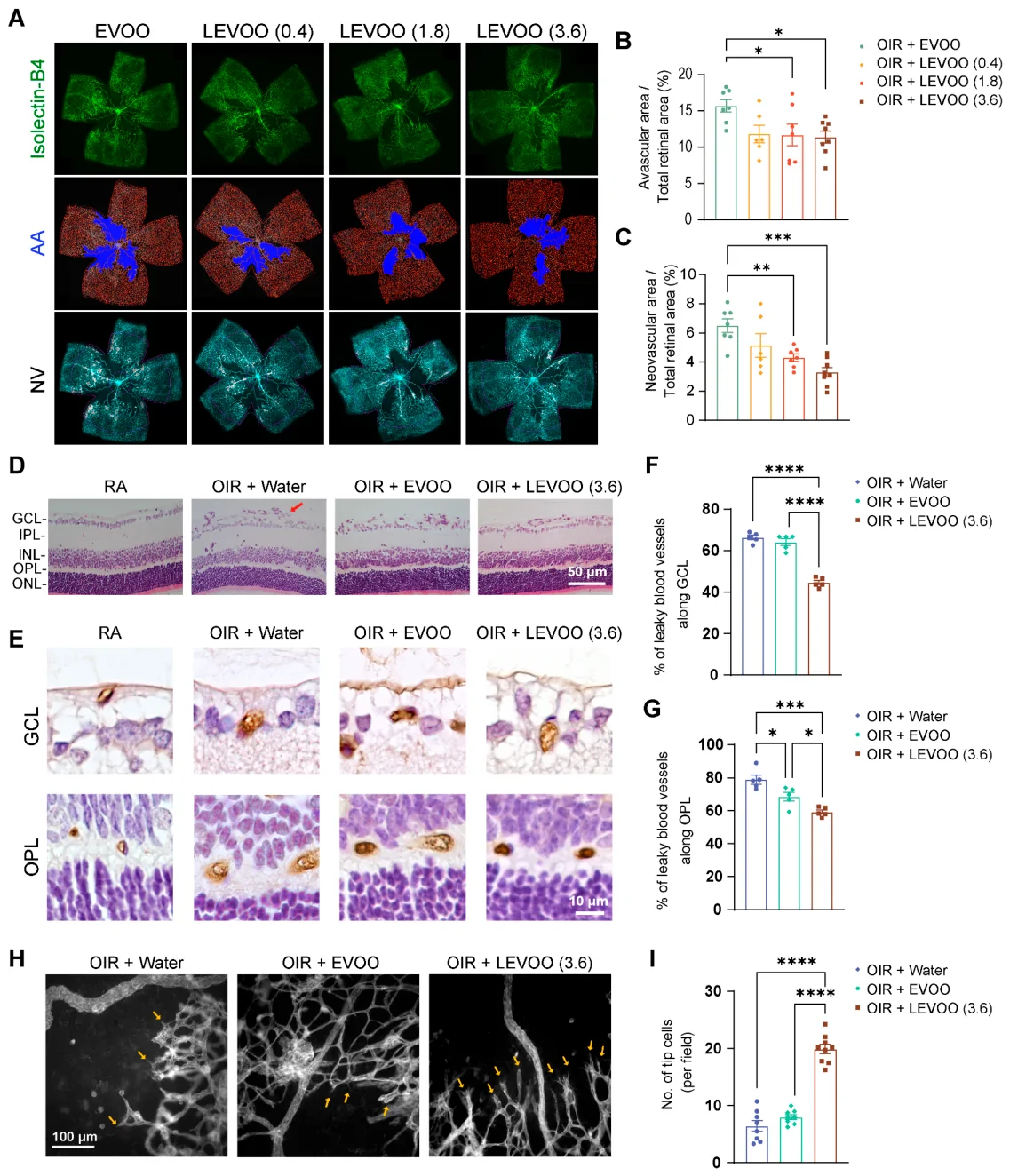
LEVOO administration improved vascular health in OIR mice. (**A**) Representative images of retina flat mounts stained with isolectin B4. The avascular area (AA) was marked in blue and the neovascular area (NV) was in white. (**B**,**C**) Quantitative analysis of AA and NV areas (*n* = 6–8; one-way ANOVA followed by Dunnett’s test). (**D**) H&E staining of retinal sagittal sections. The red arrow indicates the neovascular tufts. Scale bar, 50 μm. (**E**–**G**) IHC staining and quantitative analysis of IgG extravasation in the vessels in GCL and OPL (*n* = 5; one-way ANOVA followed by Tukey’s test). Diffuse IgG staining was observed outside the vascular lumen, indicating leaky vessels in OIR. Scale bar, 10 μm. (**H**,**I**) Representative images and quantitative analysis of endothelial tip cells (yellow arrow) (*n* = 8–9; one-way ANOVA followed by Tukey’s test). Scale bar, 100 μm.

LEVOO administration with lutein at 1.8 μg/g or 3.6 μg/g resulted in a significantly smaller AA than in EVOO-treated controls (Figure 3A,B). Furthermore, LEVOO induced a dose-dependent reduction in NV, with 1.8 μg/g and 3.6 μg/g yielding significant decreases relative to EVOO alone (Figure 3A,C). The attenuation of pathological neovascular tufts by LEVOO was corroborated by H&E staining (Figure 3D).

As blood-retinal barrier (BRB) disruption is a characteristic feature in OIR, we evaluated IgG extravasation. Vessel leakage was defined by IgG immunostaining detected outside the vascular lumen (Figure 3E) [33,36]. Quantification revealed that LEVOO supplementation, but not EVOO alone, significantly reduced the percentage of leaky vessels in the ganglion cell layer (GCL) (Figure 3F). EVOO supplementation reduced vessel leakage in the outer plexiform layer (OPL), while LEVOO administration further decreased the percentage of leaky vessels (Figure 3G).

To investigate the potential promotion of normal vessel growth, we analyzed endothelial tip cells in the superficial vascular plexus in the transition zone between the avascular and vascularized areas. Tip cells guide the development of vascular sprouts and are critical for angiogenesis [37]. LEVOO supplementation (3.6 μg/g/day) significantly increased the number of tip cells (Figure 3H,I), suggesting that LEVOO could promote tip cell formation and subsequent normal vascular development in the transition zone.

Collectively, these results demonstrated that LEVOO improved vascular health by inhibiting pathological angiogenesis, fostering BRB integrity, and promoting vessel growth in the avascular area in the OIR model.

### 3.4. LEVOO Administration Improved Retinal Function and Protected Neurons upon OIR

**Figure 4:**
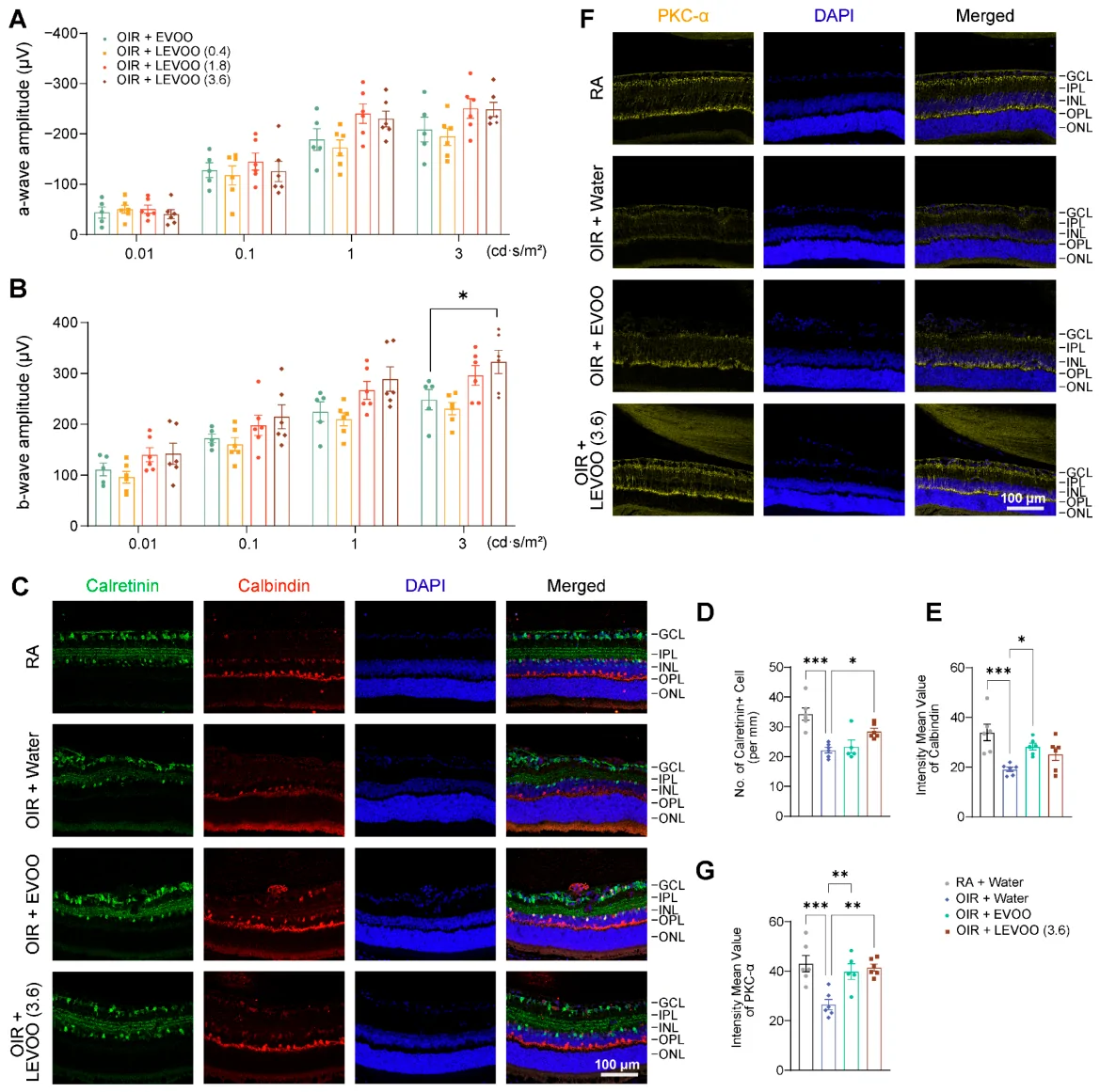
LEVOO administration improved retinal function and preserved amacrine cells and rod bipolar cells upon OIR. (**A**,**B**) Analysis of the a-wave and b-wave amplitudes from scotopic ERG waveforms measured at the light intensities of 0.01, 0.1, 1 and 3 cd·s/m^2^ (*n* = 5–6; one-way ANOVA followed by Dunnett’s test). (**C**,**F**) Representative IF images of calretinin (green), calbindin (red) (**C**) and PKC-α (yellow) (**F**) in the retina of OIR mice at P17. (**D**) Quantification of calretinin-positive cells in the INL. (**E**,**G**) Semi-quantification of IF intensity of calbindin (**E**) and PKC-α (**G**) (*n* = 5–6; one-way ANOVA followed by Tukey’s test). Scale bar, 100 μm.

Retinal function was assessed using ERG. LEVOO-treated mice reared in RA displayed comparable a-wave and b-wave amplitudes as EVOO-treated controls (Figure S1), suggesting that LEVOO did not impair retinal function in normal neonatal mice. No significant differences in a-wave amplitudes were detected between LEVOO- and EVOO-treated mice upon OIR (Figure 4A). However, LEVOO treatment induced a dose-dependent rise in b-wave amplitudes under the tested light intensities. Specifically, mice receiving LEVOO at 3.6 μg/g exhibited significantly larger b-wave amplitudes (Figure 4B). These results demonstrated that oral LEVOO supplementation improved retinal function upon OIR. The highest LEVOO dose (3.6 μg/g of lutein) most effectively alleviated retinal vascular pathology and improved function and was thus selected for further analysis.

Given that LEVOO supplementation increased b-wave amplitudes but not a-wave amplitudes, we postulated that its protective effects might involve preservation of bipolar cells. In addition, INL is a region vulnerable to damage from the aberrant vascular development characteristic of OIR [37,38]. Therefore, various retinal neurons, including amacrine cells, horizontal cells and rod bipolar cells, in the INL were then identified by IF (Figure 4C–F). Calretinin is present in specific subcategories of amacrine cells and retinal ganglion cells. As reported previously, the OIR challenge markedly reduced the count of calretinin-positive amacrine cells (Figure 4C,D) [39,40]. Notably, LEVOO supplementation diminished this loss (Figure 4C,D). Additionally, calbindin immunoreactivity, which indicated the horizontal cells, significantly decreased in the retina of OIR mice. EVOO treatment effectively mitigated this decrease, while LEVOO showed a non-significant trend towards restoring calbindin immunoreactivity (Figure 4C,E). A notable decrease in protein kinase C alpha (PKC-α) immunoreactivity in the retina of OIR mice was observed, suggesting reduced reactivity of rod bipolar cells. Both EVOO and LEVOO administration significantly reversed this effect (Figure 4F,G). Together, these results indicated that LEVOO administration restored amacrine cells and rod bipolar cells, exerting neuroprotective effects in OIR.

### 3.5. Oral Supplementation of LEVOO Alleviated Oxidative Stress and Inflammation Caused by OIR Challenge

**Figure 5:**
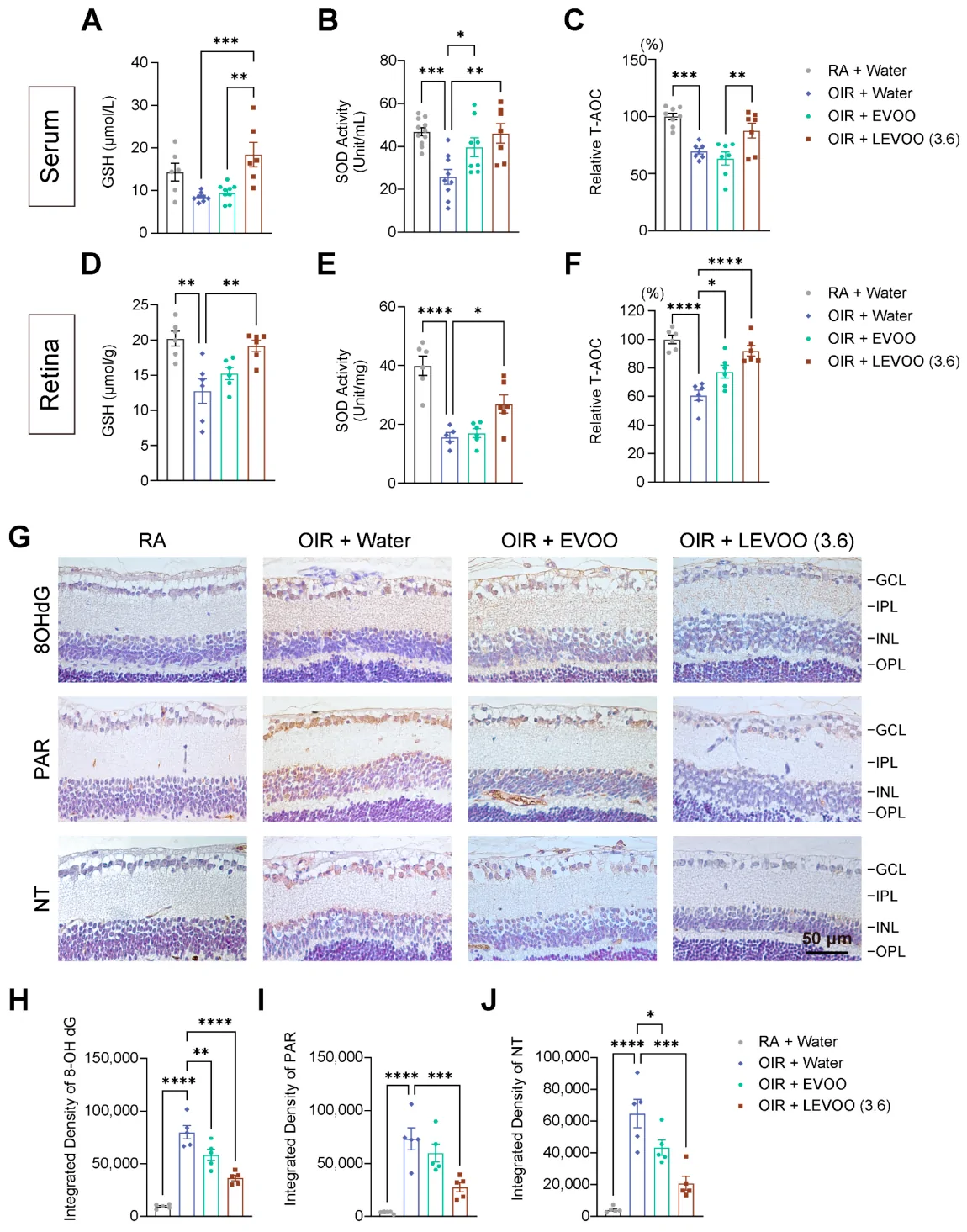
Oral supplementation of LEVOO reduced oxidative stress caused by OIR. (**A**–**C**) Serum levels of reduced GSH, SOD activity and relative T-AOC (*n* = 6–11). (**D**–**F**) Retinal levels of reduced GSH, SOD activity and relative T-AOC (*n* = 5–6). (**G**) Representative images of IHC staining and (**H**–**J**) semi-quantification of IHC scores of 8-OHdG, PAR and NT immunoreactivity (*n* = 5). One-way ANOVA followed by Tukey’s test. Scale bar, 50 μm.

OIR challenge markedly reduced serum levels of SOD activity and T-AOC and showed a trend of decreased levels of serum GSH (Figure 5A–C). Similarly, retinal levels of GSH, SOD and T-AOC were reduced in the OIR retina (Figure 5D–F). LEVOO administration upregulated the levels of these systemic and local antioxidant indicators, confirming its antioxidative capacity (Figure 5A–F). Cellular oxidative damage in retinal tissue was assessed by IHC, using biomarkers for oxidative stress-mediated injury (Figure 5G). 8-Hydroxy-2′-deoxyguanosine (8-OHdG) and poly(ADP-ribose) (PAR) were markers of DNA damage, and nitrotyrosine (NT) indicated protein nitration. OIR challenge markedly upregulated the expression of all three markers, which was diminished by LEVOO supplementation (Figure 5H–J). The results suggested that LEVOO effectively ameliorated OIR-induced oxidative stress by enhancing systemic and retinal redox defense and attenuating the oxidative stress-mediated damage in the retina.

**Figure 6:**
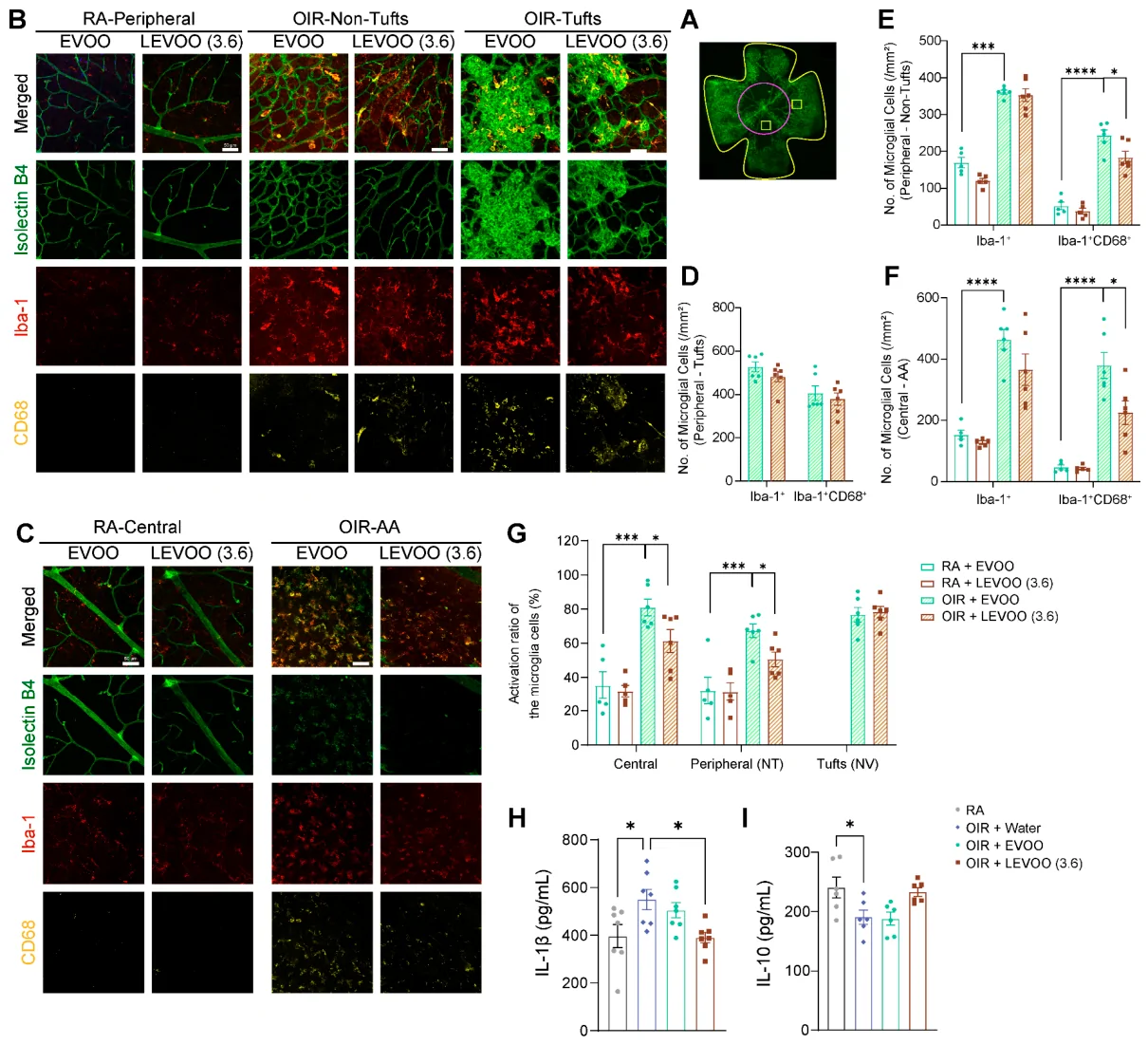
Oral administration of LEVOO alleviated inflammatory responses elicited by OIR. (**A**) Schematic illustration of the central and peripheral regions in a retinal flat mount. (**B**,**C**) Representative confocal images of microglia in the peripheral (**B**) and central (**C**) retina. (**D**–**G**) Quantification of microglia (Iba-1^+^) and activated microglia (Iba-1^+^CD68^+^) and the ratio of activated microglia cells (*n* = 5–6; unpaired *t*-test). (**H**,**I**) Levels of IL-1β (**H**) and IL-10 (**I**) in the retina (*n* = 5–6; one-way ANOVA followed by Tukey’s test). Scale bar, 50 μm.

We next assessed the inflammatory status of the retina in OIR mice. Retinal microglia were labeled and analyzed (Figure 6A–C). Total microglia were labeled with ionized calcium-binding adapter molecule 1 (Iba-1), and activated microglia were identified by cluster of differentiation 68 (CD68) co-localization. Confocal images were obtained in the central avascular area and peripheral regions with or without neovascular tufts. Quantification revealed a dramatic increase in microglia (Iba-1^+^) and activated microglia (Iba-1^+^CD68^+^) numbers, as well as the activation ratio in the retina of OIR mice compared with RA-reared mice. Oral LEVOO administration significantly reduced the number of activated microglia and the activation ratio in the central avascular area and in non-tuft peripheral areas (Figure 6D–G).

ELISA analysis revealed that OIR upregulated the pro-inflammatory cytokine IL-1β, which was suppressed by LEVOO treatment (Figure 6H). Levels of the anti-inflammatory IL-10 decreased upon OIR. LEVOO supplementation showed a trend towards increasing IL-10 levels, but without a statistically significant difference (Figure 6I). These results indicated that LEVOO effectively attenuated neuroinflammation in OIR by reducing microglial activation and suppressing IL-1β.

### 3.6. Proteomic Findings Provided Mechanistic Insights into LEVOO’s Benefits

**Figure 7:**
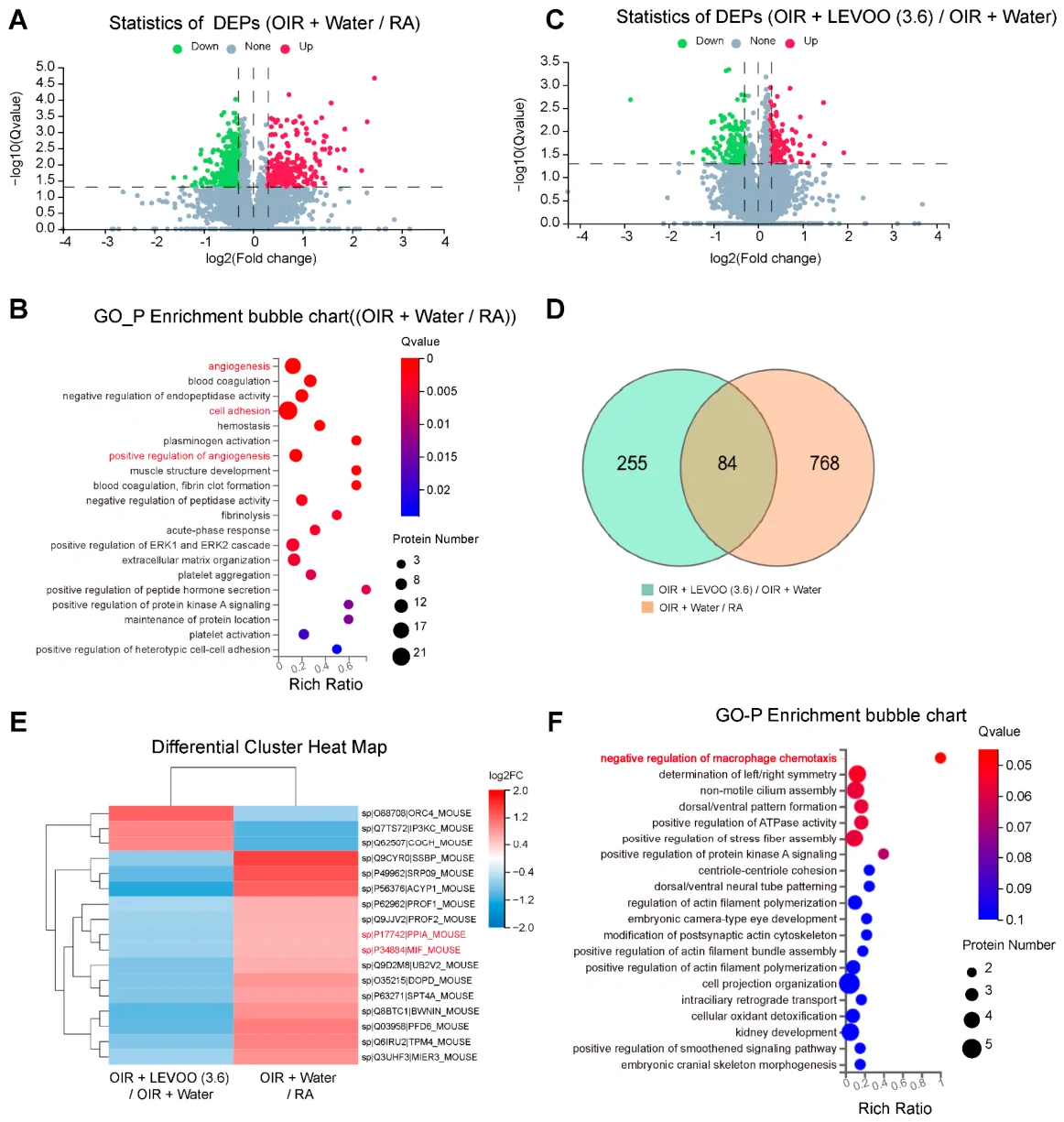
Proteomic analysis provided mechanistic insights into LEVOO’s benefits. (**A**,**B**) Volcano plots showing the significant DEPs (FC > 1.2; Q < 0.05) of RA-reared mice compared with OIR-challenged mice (**A**) and of OIR-challenged mice fed with LEVOO at 3.6 μg/g/day of lutein compared with those fed water (**B**). (**C**) GO enrichment analysis (showing the top 20 biological processes) of DEPs in the OIR group compared with the RA group. (**D**) Venn diagram showing the number of DEPs in common (FC > 1.2; Q < 0.05). (**E**) The differential cluster heat map showing the DEPs (top 17) in two comparisons. (**F**) GO enrichment analysis (showing the top 20 biological processes) of the 84 DEPs in common.

Building on these consistently beneficial effects, we used proteomic analysis to explore the underlying mechanisms. There were 274 upregulated and 605 downregulated differentially expressed proteins [DEPs; fold change (FC) > 1.2, Q < 0.05] between the retinas of OIR mice and RA controls (Figure 7A). Gene Ontology (GO) enrichment analysis of these DEPs identified enrichment in biological processes, including angiogenesis and blood coagulation (Figure 7B).

We next identified proteins modulated by LEVOO treatment. Comparison of LEVOO-treated OIR mice versus those treated with water identified 171 upregulated and 168 downregulated DEPs in the retina (FC > 1.2, Q < 0.05) (Figure 7C). Among them, 84 DEPs were common in both the OIR-induced and LEVOO-modulated proteomes (Figure 7D), representing key targets of the intervention. A hierarchical cluster heatmap of these 84 shared DEPs revealed that proteins such as peptidylprolyl isomerase A (PPIA; alternatively termed cyclophilin A, CypA) and macrophage migration inhibitory factor (MIF) were upregulated by OIR challenge but normalized toward levels in RA-reared mice by LEVOO treatment (Figure 7E). GO enrichment analysis of the 84 LEVOO-modulated DEPs in the retina of OIR mice further indicated that LEVOO supplementation negatively regulated macrophage chemotaxis (Figure 7F), suggesting that LEVOO might engage a pivotal therapeutic role via suppressing the recruitment of inflammatory cells to the retina.

### 3.7. Oral Supplementation of LEVOO Inhibited the HIF-1α/MIF Signaling and the LRP1/CypA/MMP9 Axis

**Figure 8:**
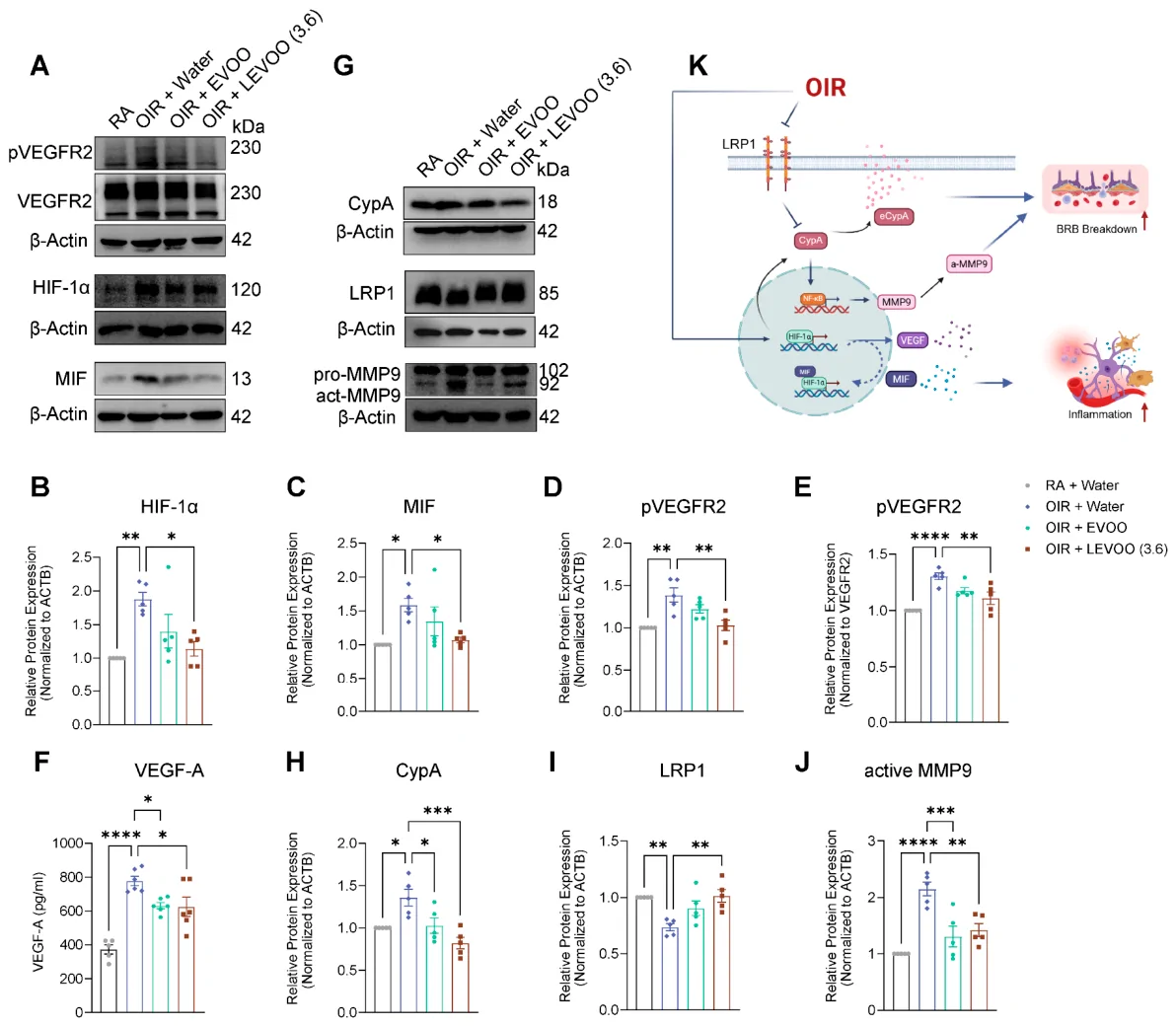
Oral supplementation of LEVOO inhibited the HIF-1α/MIF signaling and the LRP1/CypA/MMP9 signaling. (**A**,**G**) Western blotting assay and (**B**–**E**,**H**–**J**) quantification of relative expression levels for the corresponding proteins: HIF-1α, MIF, *p*-VEGFR2, VEGFR2, CypA, LRP1 and active MMP9. (**F**) Retinal levels of VEGF-A analyzed by ELISA. (*n* = 5; one-way ANOVA followed by Tukey’s test). (**K**) Schematic illustration of the signalling in the OIR retina.

MIF was reported to be proangiogenic and proinflammatory in retinal neovascularization and was redundantly regulated by HIF-1α [41,42,43]. The HIF-1α/VEGF-A/VEGF receptor 2 (VEGFR2) signaling is crucial in pathological neovascularization and was upregulated in OIR [44]. Our WB and ELISA analyses confirmed that this pathway was activated, and MIF was upregulated in OIR. Notably, LEVOO supplementation significantly reduced the levels of HIF-1α, MIF, and phosphorylated VEGFR2 (*p*-VEGFR2) and VEGF-A (Figure 8A–F).

CypA was identified as a mediator of angiogenesis and blood–brain barrier (BBB) disruption [45,46]. As indicated in proteomic data, our WB analysis confirmed that OIR challenges upregulated CypA expression, which was suppressed by LEVOO supplementation (Figure 8G,H). The low-density lipoprotein receptor-related protein-1 (LRP1)/CypA/matrix metalloproteinase-9 (MMP9) pathway was reported to be involved in BBB disruption in apolipoprotein E-related cognitive decline and subarachnoid hemorrhage [47,48]. We therefore further evaluated the expression of LRP1, MMP9, and the active form of MMP9, a direct effector of BRB breakdown [49]. WB analysis indicated that OIR challenge downregulated LRP1 expression and upregulated the active MMP9, which were reversed by LEVOO supplementation (Figure 8G,I,J). Together, these results suggested that oral LEVOO administration inhibited the HIF-1α/MIF signaling and the LRP1/CypA/MMP9 axis in the OIR retina (Figure 8K).

### 3.8. Oral Supplementation of LEVOO Helped to Balance Fatty Acid Components and Restore Dopamine Levels in the Retina of OIR Mice

**Figure 9:**
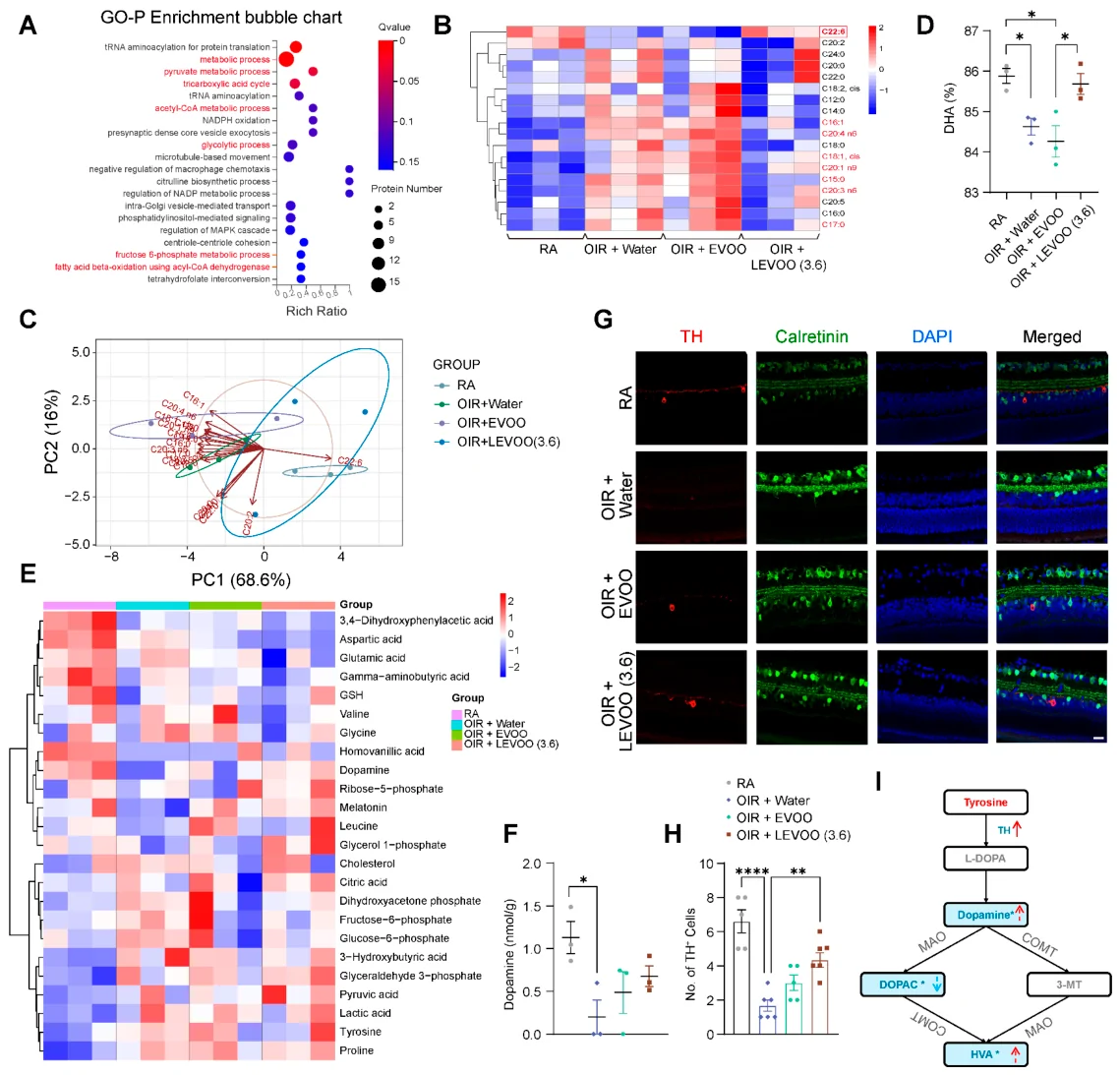
Oral supplementation of LEVOO helped to balance fatty acid disorder and restore retinal dopamine in the retina of OIR mice. (**A**) GO enrichment showing the top 20 biological processes analyzed from all DEPs in common (OIR-LEVOO/OIR-Water and OIR-Water/RA; FC > 1; Q < 0.05). (**B**) The heatmap for the C4:0–C26:0 fatty acids percentage among 4 groups (*n* = 3). The significantly changed fatty acids were indicated in red (*p* < 0.05, one-way ANOVA). (**C**) PCA biplot of the retinal fatty acid components. (**D**) Scatter plot showing the percentage of DHA among the fatty acids. (**E**) Heatmap of average levels of selected metabolites (*n* = 3). (**F**) Dopamine levels in the retina among four groups. (**G**) Representative images of IF staining of calretinin (green) and TH (red) in the retina. Scale bar, 20 μm. (**H**) Quantification of the TH-positive amacrine cells in the innermost layer of INL (*n* = 5–6; one-way ANOVA followed by Tukey’s test). (**I**) Diagram of retinal dopamine metabolism changes in OIR versus RA, with increases/decreases marked in red/blue text, respectively (those with * indicating a *p* < 0.05). Effects of LEVOO treatment after OIR were shown with red/blue arrows (solid for *p* < 0.05). Unchanged or unidentified intermediates were in gray. (3-MT: 3-O-methyldopa, DOPAC: 3,4-dihydroxyphenylacetic acid, L-DOPA: 3,4-dihydroxyphenylalanine, COMT: catechol O-methyltransferase, HVA: homovanillic acid, MAO: monoamine oxidase; TH: tyrosine hydroxylase).

Metabolism plays a crucial role in the pathogenesis of retinal neovascularization [50,51]. GO enrichment analysis of all DEPs (FC > 1, Q < 0.05) indicated that LEVOO administration influenced biological processes such as the pyruvate metabolic process and tricarboxylic acid cycle (Figure 9A). To evaluate retinal metabolite levels, targeted GC-MS/MS was used to analyze selected metabolites, including polar metabolites (amino acids, central carbon metabolites, sugars, neurotransmitters) and fatty acids (C4:0–C26:0) among four groups: RA controls and OIR-challenged mice treated with water, EVOO, or LEVOO (3.6 μg/g of lutein).

Assessment suggested that OIR challenge and LEVOO supplementation altered the retinal fatty acid composition (Figure 9B). To obtain a holistic view of variations, a PCA biplot was constructed, which implied that docosahexaenoic acid (DHA, C22:6) was the primary driving variable in principal component 1 (Figure 9C). Given that DHA constituted a dominant proportion (over 80%) of the total fatty acid content in the samples, its fluctuations had the most influence on the overall remodeling of the fatty acid profile. Therefore, we focused on DHA. Evaluation of the proportions of retinal fatty acids revealed a significant reduction in DHA induced by OIR challenge. Remarkably, DHA levels in the OIR-challenged mice fed with LEVOO tended to be higher compared with those fed with water and were significantly higher than those fed with EVOO alone (Figure 9D).

For polar metabolites, the OIR challenge caused a significant decrease in retinal dopamine levels, which was partially reversed by LEVOO supplementation (Figure 9E,F). A similar recovery trend was observed for homovanillic acid, a primary dopamine metabolite (Figure 9E). The biosynthesis of dopamine is initiated by tyrosine hydroxylase (TH), the rate-limiting enzyme. Co-immunostaining with antibodies against calretinin and TH revealed a reduction in TH-positive amacrine cells in the retina of OIR mice, which was reversed by LEVOO supplementation (Figure 9G,H). These findings suggested that LEVOO restored the TH-positive amacrine cell population, which is critical for retinal development and dopaminergic signaling (Figure 9I).

## 4. Discussion

**Figure 10:**
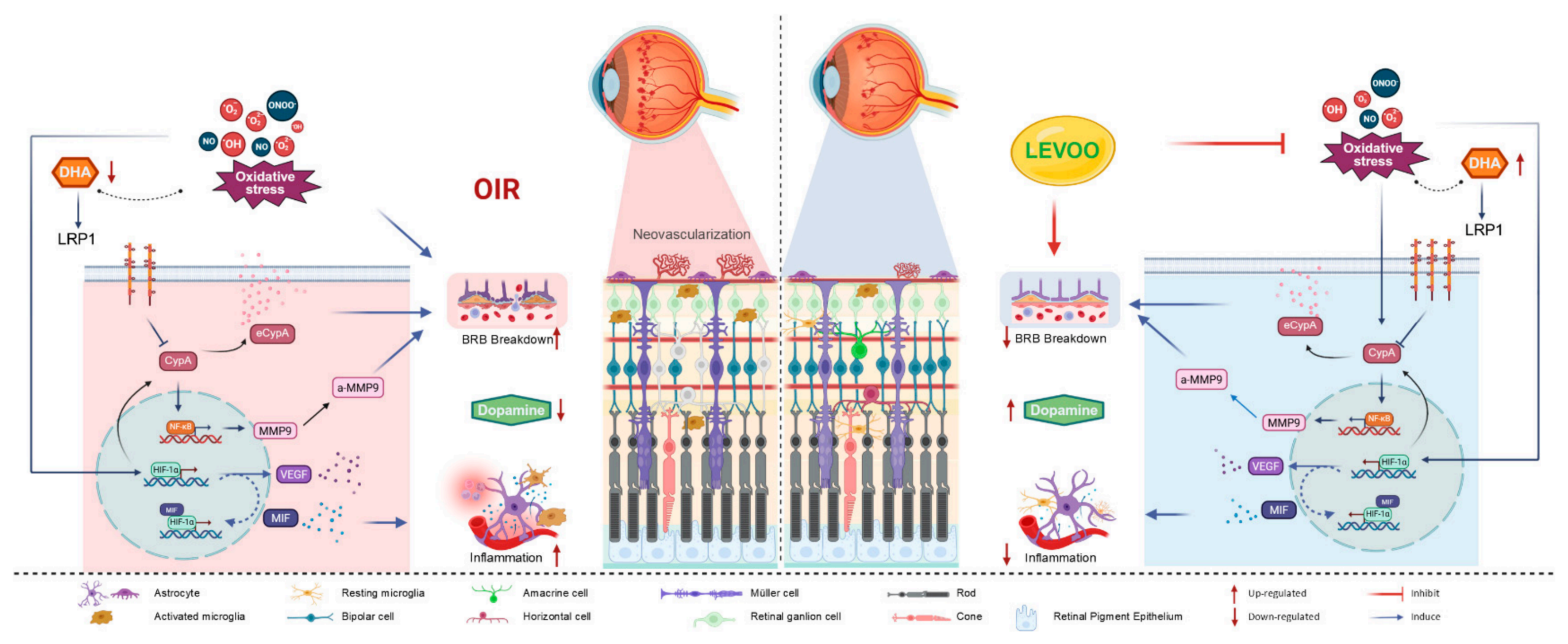
Schematic illustration of the actions of LEVOO in OIR and its underlying mechanisms. Supplementation of LEVOO alleviated the OIR-induced damage by counteracting oxidative stress and alleviating inflammation, protecting BRB integrity, modulating the HIF-1α/MIF and LRP1/CypA/MMP9 signaling, and restoring retinal DHA and dopamine levels. (eCypA: extracellular CypA; a-MMP9: active MMP9; NF-κB: nuclear factor kappa B). This graph was generated via BioRender.

Our findings established LEVOO’s safety profile in neonatal mice and demonstrated its multifaceted benefits following OIR-induced retinal injury. LEVOO supplementation inhibited pathological neovascularization, promoted reparative vascular growth, and improved retinal function. Oral LEVOO supplementation can partially restore retinal levels of DHA and dopamine, which are important for retinal structure and function (Figure 10). Notably, EVOO alone also showed trends toward retinal benefit, suggesting that EVOO may contribute to the observed effects beyond acting as a delivery vehicle in the OIR model.

In a normally developing mouse retina, vascular branches from the superficial plexiform plexus extend toward the deep plexus, reaching the outer edge of the INL by P12, thereby ensuring sufficient nutrient delivery to retinal neurons [52]. In OIR, this process was disrupted, leading to relative ischemia in the INL. Our previous and current studies demonstrated a significant reduction in INL neurons, including bipolar cells, in OIR-affected retinas [31]. Consistent with this, a dose-dependent increase in the dark-adapted b-wave amplitude, which is primarily generated by rod-driven ON bipolar cells, was shown in LEVOO-treated mice [38]. These results suggested that LEVOO coordinately improved the retinal vasculature and benefited the neurons and retinal function.

Oxidative stress and inflammation exerted widespread effects in ROP babies and the OIR model [53,54]. Increased microglial activation was previously observed in the OIR-affected retina, and modulating the microglial/macrophage transition toward the anti-inflammatory phenotype could alleviate OIR-induced damage [53]. The antioxidative and anti-inflammatory properties of EVOO and lutein have been well documented [11,55]. In the present study, we further demonstrated that LEVOO increased retinal GSH levels, SOD activity, and T-AOC and decreased activated microglia cells directly in retinal tissues. These findings reinforced the dual antioxidative and anti-inflammatory benefits of the LEVOO formulation in the OIR model.

Beyond these established antioxidant and anti-inflammatory effects, we identified new insights into the mechanisms of LEVOO in protecting the retina from OIR, including modulation of the HIF-1α/MIF axis and LRP1/CypA/MMP9 axis and the restoration of DHA and dopamine levels.

During the relative hypoxia phase of OIR, HIF-1α expression is stimulated [44]. HIF-1α can induce the transcription of MIF, a pleiotropic proinflammatory and proangiogenic cytokine [41,56]. MIF was recognized as a key driver of pathological retinal and choroidal neovascularization. Clinical studies have reported elevated MIF levels in the vitreous and aqueous humor of patients with proliferative diabetic retinopathy (PDR) [56,57,58]. MIF could upregulate VEGF and promote migration and proliferation of retinal microvascular endothelial cells [56]. MIF can also be neurotoxic, as its inhibition was shown to be protective in both excitotoxic injury and retinal detachment models [59]. In this study, LEVOO supplementation significantly attenuated MIF upregulation upon OIR. This inhibitory effect on MIF provided a plausible mechanism for the anti-inflammatory, anti-angiogenic and neuroprotective effects of LEVOO. It was reported that MIF could in turn stabilize the HIF-1α protein and its signaling in certain hypoxic cell types, suggesting a potential positive feedback loop [60,61,62,63]. However, this regulatory loop appeared to be cell-specific [41]. Therefore, how HIF-1α/MIF signaling mediates the retinal microenvironment, particularly in OIR, requires further elucidation.

CypA is primarily known as the intracellular receptor for the immunosuppressant cyclosporine A [64]. Its expression was upregulated under hypoxic conditions. Triggered by oxidative stress, it could function as a growth factor and proinflammatory cytokine that activated endothelial cells [65,66]. In the brain, activation of the CypA/MMP9 pathway was reported to contribute to the blood–brain barrier disruption [47]. Meanwhile, endothelial cell-specific depletion of LRP1 resulted in increased retinal neovascularization in OIR [67]. Here, we reported that OIR challenge decreased LRP1 expression and upregulated the CypA/MMP9 signaling, whereas LEVOO supplementation significantly reversed these changes. These findings may explain how LEVOO administration protected BRB integrity and inhibited pathological angiogenesis in OIR, suggesting that LRP1 and CypA could be promising therapeutic targets for ROP and other pathological retinal neovascularization conditions. Further development of interventions modulating this signaling could open new avenues for clinical management.

DHA is a key lipid for retinal structure, homeostasis, and phototransduction [68]. Recent studies have linked fatty acid metabolism to the pathogenesis of ROP, and DHA supplementation has been proposed to mitigate ROP [51,69,70]. However, clinical studies have yielded inconsistent results. Meta-analyses and the 2024 Joinville DHA trial found no significant benefits of DHA in ROP [71,72,73]. In fact, DHA supplementation was observed to increase the risk of bronchopulmonary dysplasia [72]. Yet, a recent secondary analysis reported that supplementing DHA enterally with arachidonic acid (ARA) reduced the chance of developing severe ROP by 50% [74]. This study highlighted the importance of a combination of DHA and ARA rather than single DHA supplementation. However, ARA metabolism plays a complex and context-dependent role in ROP, as it is a precursor for both pro-inflammatory and pro-resolving lipid mediators [75,76]. Therefore, supplementing DHA with ARA may lead to unpredictable and unregulated consequences on disease progression, given the strong inflammatory conditions of ROP. In our study, LEVOO supplementation was shown to partially restore the retinal DHA levels in OIR mice. Likewise, lutein supplementation was found to reduce DHA oxidation in the prefrontal cortex of rhesus monkeys [77]. We postulate that lutein in LEVOO may protect endogenous DHA from oxidative destruction during OIR. LEVOO supplementation offers a safer strategy than direct DHA intake, which is associated with a higher risk of bronchopulmonary dysplasia, or co-supplementation with ARA, which carries uncertain effects. Additionally, dietary DHA supplementation has been documented to upregulate LRP1 expression in mice [78]. We therefore postulate that the increase in retinal DHA levels induced by LEVOO supplementation in the OIR model may be mechanistically linked to the LRP1/CypA signaling, although further investigations are required to elucidate the precise molecular interplay.

Dopamine is an important neurotransmitter in the vertebrate retina, where it coordinates light adaptation, regulates neural circuits and modulates ocular growth [79,80,81]. Retinal dopamine is synthesized and released by dopaminergic amacrine cells [82]. In diabetic rodent models, lack of dopamine could cause early visual impairment, while treatment with dopamine’s precursor levodopa reversed this dysregulation [83,84]. Exogenous levodopa also exerted protective effects against age-related macular degeneration by regulating dopaminergic signaling, promoting optic nerve regeneration and safeguarding vision [85,86]. In our study, retinal dopamine levels were reduced in OIR-affected retinas and partially restored by LEVOO supplementation. This finding is consistent with previous evidence that lutein protected the dopaminergic neurons in the brain, potentially relieving Parkinson’s disease in animal models [87,88]. The results established the first direct link between the nutritional intervention of LEVOO supplementation and the restoration of key neurotransmitter homeostasis in OIR, extending beyond lutein’s antioxidative and anti-inflammatory mechanisms. It suggested that LEVOO may promote retinal functional recovery partially by restoring dopaminergic amacrine cells at the neuroprotective and neurovascular-coupling levels. Future research exploring specific molecular targets of LEVOO in dopaminergic pathways may open new avenues for neuromodulator-based therapeutic strategies.

This study offers an advantageous strategy for delivering lutein, a natural but highly lipophilic carotenoid with low bioavailability. Current approaches to enhance lutein’s bioavailability primarily involve patented nano-emulsion preparations or polymer-based nanocarrier formulations, but with trade-offs such as increased costs, complex formulations, or inclusion of non-natural ingredients [89,90,91]. In contrast, the formulation of lutein-in-EVOO used in this study may not only utilize EVOO’s natural lipids to promote lutein dissolution and absorption but also offer the benefits of natural composition, low cost, and simple preparation. Furthermore, LEVOO served as a vegetarian-friendly carrier with excellent safety profiles. Given the widespread acceptance and well-tolerated nature of oil-based emulsions in premature babies, LEVOO holds clinical potential as a nutritional intervention to prevent or to adjunctively treat neonatal retinal disorders such as ROP. Its application could be explored as oral drops or as an additive in formula nutrition. The EVOO-based delivery strategy tested here may also be applicable to zeaxanthin, another important xanthophyll in the macula. Beyond ROP, LEVOO may benefit patients with other ischemic retinopathies involving retinal neovascularization, such as diabetic retinopathy.

Several limitations of this study should be acknowledged. First of all, this is a preclinical study using mice, which cannot completely replicate the complex pathologies seen in human ROP. Unlike humans, mice lack the macula in their eyes; therefore, they are not a perfect anatomical model. Secondly, the current study cannot determine whether the observed effects were due to the additive effects of lutein and EVOO, enhanced lutein bioavailability in the LEVOO formulation, or both. Previous evidence indirectly supported that olive oil enhanced lutein absorption [19], and our observation that LEVOO provided greater retinal benefit than EVOO alone is consistent with this possibility, but these data do not directly demonstrate either mechanism. Future studies are needed to determine whether EVOO improves lutein bioavailability in OIR mice and whether EVOO and lutein act additively. A lutein-in-regular-olive-oil comparator or lutein with polyphenol-depleted EVOO may help elucidate this problem. Thirdly, direct quantification of lutein in the retina was not performed, making it unclear whether lutein can reach the retina to exert direct effects. However, BRB disruption in the OIR model may theoretically allow LEVOO-derived lutein and EVOO components to reach the retina. We also acknowledge that delivery may be more limited when the BRB is intact. Fourthly, the roles of the HIF-1α/MIF and LRP1/CypA/MMP9 signaling pathways in the retinal microenvironment, particularly in the OIR model, require further elucidation. Whether these targets are direct effectors causally involved or merely an associated event in LEVOO’s mechanism of action remains to be determined. To address this, future experiments should be designed to validate causality. Finally, the mechanistic relationship between DHA modulation and the antioxidative effects of LEVOO, as well as the specific molecular targets of LEVOO in dopaminergic pathways, warrants further investigation.

## 5. Conclusions

In conclusion, our findings demonstrated that LEVOO provided multifaceted benefits in the OIR model. LEVOO supplementation inhibited pathological neovascularization, promoted reparative vascular growth, and improved retinal function. These benefits were mediated through its potent antioxidative and anti-inflammatory properties, which restored systemic and retinal redox balance, attenuated microglial activation, and suppressed key inflammatory cytokines. Notably, LEVOO partially restored retinal levels of critical metabolites, including DHA and dopamine, and preserved the neuronal populations responsible for dopamine’s synthesis. Mechanistically, these effects involved the downregulation of pivotal proteins, including CypA and MIF, suggesting novel pathways by which LEVOO mitigated vascular pathology and inflammation. Our study demonstrated LEVOO as a promising nutritional intervention for ischemic retinopathies.

## Data Availability

The original contributions presented in this study are included in the article/Supplementary Materials. The proteomic data were deposited in the ProteomeXchange Consortium (PXD085879). Further inquiries can be directed to the corresponding author.

## Supplementary Materials

The following supporting information can be downloaded at https://www.mdpi.com/article/10.3390/nu18213616/s1, Table S1: The antibodies used in this study; Table S2: The assay kits used for measuring serum biochemical and oxidative stress markers, and retinal oxidative stress markers and inflammatory cytokines; Figure S1: LEVOO did not impair retinal function in mouse neonates raised in room air. (**A**–**D**) Representative ERG waveforms measured by scotopic ERG at the light intensity of 0.01 cd·s/m^2^ (**A**), 0.1 cd·s/m^2^ (**B**), 1 cd·s/m^2^ (**C**) and 3 cd·s/m^2^ (**D**) of mice raised in room air at P17 receiving different supplementations. (**E**,**F**) Bar plots showing the a-wave amplitudes (**E**) and b-wave amplitudes (**F**). (*n* = 6; One-way ANOVA followed by Tukey’s post hoc test, no statistical differences were found).

## Abbreviations

8-OHdG	8-hydroxy-2′-deoxyguanosine
AA	Avascular area
ALT	Alanine aminotransferase
ARA	Arachidonic acid
AST	Aspartate aminotransferase
BRB	Blood-retinal barrier
BUN	Blood urea nitrogen
CD68	Cluster of Differentiation 68
CRE	Creatinine
CypA	Cyclophilin A
DEPs	Differentially expressed proteins
DHA	Docosahexaenoic acid
EVOO	Extra-virgin olive oil
FC	Fold change
GCL	Ganglion cell layer
GFAP	Glial fibrillary acidic protein
GO	Gene ontology
GSH	Reduced glutathione
H&E	Hematoxylin and Eosin
HIF-1α	Hypoxia-inducible factor-1α
Iba-1	Ionized calcium-binding adapter molecule 1
IF	Immunofluorescence
IHC	Immunohistochemical
IL	Interleukin
INL	Inner nuclear layer
LEVOO	Lutein-in-EVOO
LRP1	Low-density lipoprotein receptor-related protein-1
MIF	Macrophage migration inhibitory factor
MMP9	Matrix metalloproteinase-9
NT	Nitrotyrosine
NV	Neovascular area
OIR	Oxygen-induced retinopathy
OPL	Outer plexiform layer
OO	Olive oil
PAR	Poly(ADP-ribose)
PCA	Principal component analysis
PKC-α	Protein kinase C alpha
RA	Room air
ROP	Retinopathy of prematurity
ROS	Reactive oxygen species
SOD	Superoxide dismutase
T-AOC	Total antioxidant capacity
T-CHO	Total cholesterol
TG	Triglycerides
TH	Tyrosine hydroxylase
VEGF	Vascular endothelial growth factor
VEGFR2	VEGF receptor 2
