# Supplementary material for: Lutein-in-Extra-Virgin-Olive-Oil Supplementation as a Multifaceted Nutritional Intervention for Retinopathy of Prematurity: Evidence from the Mouse Oxygen-Induced Retinopathy Model
Source: Nutrients. 2026 Oct 30;18(21):3616. doi: 10.3390/nu18213616 (PMC13765218; doi:10.3390/nu18213616)
Supplement: Supplementary file 1 [file nutrients-18-03616-s001.zip › nutrients-4525618-supplementary/Supplementary/Supplementary.pdf]

Table S1. The antibodies used in this study.

Antibodies	Source	Catalog No.
**Immunofluorescence (IF) staining**		
Alexa Fluor-488-conjugated Griffonia simplicifolia isolectin B4 (GS-IB4)	Invitrogen, USA	I21411
Ionized calcium-binding adaptor molecule 1(Iba-1)	Wako Chemicals USA	019-19741
Cluster of Differentiation 68 (CD68)	Bio-Rad, USA	MCA1957
Protein kinase C alpha (PKC-α)	Santa Cruz Biotechnology Inc., CA, USA	SC208
Calretinin	Santa Cruz Biotechnology Inc., CA, USA	SC-11644
Calbindin	Abcam, UK	ab229915
Glutamine synthetase (GS)	Abcam, UK	ab228590
Glial fibrillary acidic protein (GFAP)	Dako, Denmark	Z0334
Tyrosine hydroxylase (TH)	Proteintech, Wuhan, China	25859-1-AP
AlexaFluor 488-conjugated secondary antibody	Thermo Fisher, USA	A11001
AlexaFluor 555-conjugated secondary antibody	Thermo Fisher, USA	A21432
AlexaFluor 568-conjugated secondary antibody	Thermo Fisher, USA	A11011
DAPI (0.1 μg/ml)	Sigma	D9564
**Immunohistochemistry (IHC) staining**		
8-hydroxy guanosine (8-OHdG)	Abcam, UK	ab10802
Nitrotyrosine (NT)	Santa Cruz Biotechnology Inc., CA, USA	sc-32757
Poly(ADP-ribose) (PAR)	Enzo Life Sciences, Farmingdale, NY, USA	ALX-804-220-R100
HRP-conjugated secondary antibody (PV-8000)	Beijing Zhongshan Golden Bridge Biological Technology Co., Ltd, Beijing, China	PV-8000
HRP-labelled donkey anti-goat secondary antibody	Beyotime, Shanghai, China	A0181
**Western Blot (WB) Analysis**		
Vascular endothelial growth factor receptor-2 (VEGFR2)	Proteintech, Wuhan, China	26415-1-AP
Phospho-VEGF Receptor 2 (Tyr1175) (pVEGFR2)	Cell Signaling Technology, USA	CST2478
Macrophage migration inhibitory factor (MIF)	Abcam, UK	ab187064
Hypoxia inducible factor 1 alpha (HIF-1α)	Proteintech, Wuhan, China	82989-4-RR
Cyclophilin A (CypA)	Proteintech, China	10720-1-AP
Matrix metallopeptidases 9 (MMP-9)	Abcam, United Kingdom	ab38898
Low-density lipoprotein receptor-related protein 1 (LRP1)	Abcam, United Kingdom	ab92544
β-actin (internal reference)	Cell Signaling Technology, USA	CST8457
HRP-labeled goat anti-rabbit secondary antibody	Beyotime, China	A0208
Goat anti-mouse secondary antibody (HRP)	Beyotime, China	A0216

Table S2. The assay kits used for measuring serum biochemical and oxidative stress markers, and retinal oxidative stress markers and inflammatory cytokines.

Product	Source	Catalog No.
Alanine aminotransferase (ALT)	Nanjing Jiancheng Corp., Nanjing, China	C009-2-1
Aspartate aminotransferase (AST)	Nanjing Jiancheng Corp., Nanjing, China	C010-2-1
Triglyceride (TG)	Nanjing Jiancheng Corp., Nanjing, China	A110-1-1
Total cholesterol (T-CHO)	Nanjing Jiancheng Corp., Nanjing, China	A111-1-1
Creatinine (CRE)	Nanjing Jiancheng Corp., Nanjing, China	C011-2-1
Serum urea nitrogen (BUN)	Nanjing Jiancheng Corp., Nanjing, China	C013-2-1
Total superoxide dismutase (SOD)	Beyotime, China	S0101M
Reduced glutathione (GSH)	Nanjing Jiancheng Corp., Nanjing, China	A006-2-1
Total antioxidant capacity (T-AOC)	Nanjing Jiancheng Corp., Nanjing, China	A015-2-1
Mouse VEGF-A ELISA Kit	Elabscience, China	E-EL-M1292
Mouse Interleukin-1β ELISA Kit	Elabscience, China	E-EL-M0037
Mouse Interleukin-10 ELISA Kit	Elabscience, China	E-EL-M0046

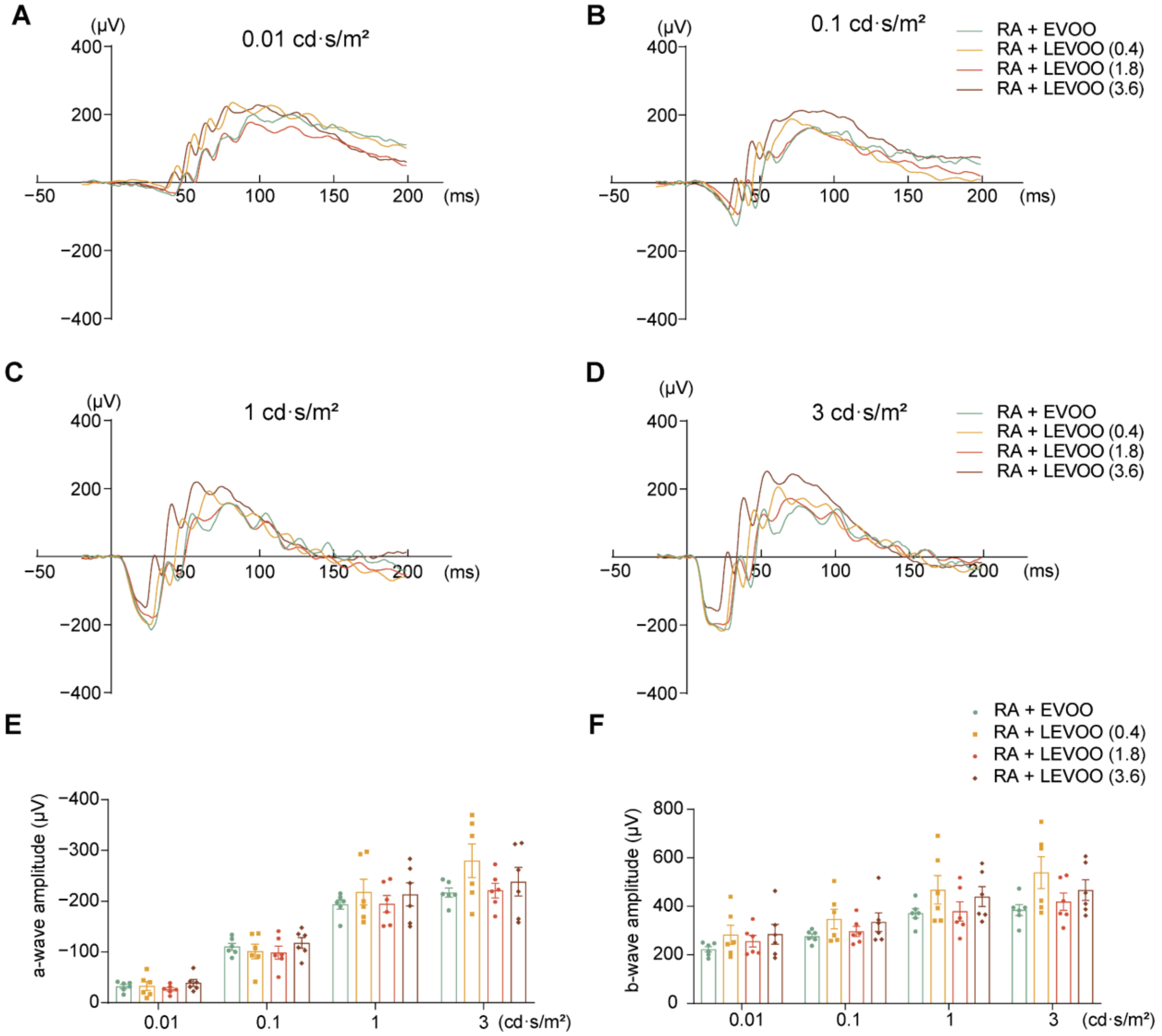

Figure S1. LEVOO did not impair retinal function in mouse neonates raised in room air. **(A-D)** Representative ERG waveforms measured by scotopic ERG at the light intensity of 0.01 cd·s/m² (A), 0.1 cd·s/m² (B), 1 cd·s/m² (C) and 3 cd·s/m² (D) of mice raised in room air at P17 receiving different supplementations. **(E, F)** Bar plots showing the a-wave amplitudes (E) and b-wave amplitudes (F). (n = 6; One-way ANOVA followed by Tukey's post hoc test, no statistical differences were found).
